# Real-time dual prediction of intradialytic hypotension and hypertension using an explainable deep learning model

**DOI:** 10.1038/s41598-023-45282-1

**Published:** 2023-10-23

**Authors:** Donghwan Yun, Hyun-Lim Yang, Seong Geun Kim, Kwangsoo Kim, Dong Ki Kim, Kook-Hwan Oh, Kwon Wook Joo, Yon Su Kim, Seung Seok Han

**Affiliations:** 1 Department of Internal Medicine, Seoul National University College of Medicine, 103 Daehak-ro, Jongno-gu, Seoul, 03080 Korea; 2 Department of Biomedical Sciences, Seoul National University College of Medicine, Seoul, Korea; 3 Department of Anesthesiology and Pain Medicine, Seoul National University Hospital, Seoul, Korea; 4 Biomedical Research Institute, Seoul National University Hospital, Seoul, Korea; 5 Department of Internal Medicine, Inje University College of Medicine, Busan, Korea; 6 Transdisciplinary Department of Medicine and Advanced Technology, Seoul National University Hospital, Seoul, Korea

**Keywords:** Haemodialysis, Machine learning

## Abstract

Both intradialytic hypotension (IDH) and hypertension (IDHTN) are associated with poor outcomes in hemodialysis patients, but a model predicting dual outcomes in real-time has never been developed. Herein, we developed an explainable deep learning model with a sequence-to-sequence-based attention network to predict both of these events simultaneously. We retrieved 302,774 hemodialysis sessions from the electronic health records of 11,110 patients, and these sessions were split into training (70%), validation (10%), and test (20%) datasets through patient randomization. The outcomes were defined when nadir systolic blood pressure (BP) < 90 mmHg (termed IDH-1), a decrease in systolic BP ≥ 20 mmHg and/or a decrease in mean arterial pressure ≥ 10 mmHg (termed IDH-2), or an increase in systolic BP ≥ 10 mmHg (i.e., IDHTN) occurred within 1 h. We developed a temporal fusion transformer (TFT)-based model and compared its performance in the test dataset, including receiver operating characteristic curve (AUROC) and area under the precision-recall curves (AUPRC), with those of other machine learning models, such as recurrent neural network, light gradient boosting machine, random forest, and logistic regression. Among all models, the TFT-based model achieved the highest AUROCs of 0.953 (0.952–0.954), 0.892 (0.891–0.893), and 0.889 (0.888–0.890) in predicting IDH-1, IDH-2, and IDHTN, respectively. The AUPRCs in the TFT-based model for these outcomes were higher than the other models. The factors that contributed the most to the prediction were age and previous session, which were time-invariant variables, as well as systolic BP and elapsed time, which were time-varying variables. The present TFT-based model predicts both IDH and IDHTN in real time and offers explainable variable importance.

## Introduction

The number of patients on hemodialysis continues to increase, reaching more than 3.9 million worldwide^1^. Hemodialysis patients have death rates that are 10 to 15 times higher than those of nondialytic controls. Cardiovascular events, such as arrhythmia and cardiac arrest, account for half of all deaths^2^, and both intradialytic hypotension (IDH) and intradialytic hypertension (IDHTN) are significant risk factors for these events^3–6^. Early warning of IDH and IDHTN can assist clinicians in preparing management strategies, and an individualized approach may be necessary due to the heterogeneity of patients and their hemodialysis settings^7,8^. Accordingly, defining risk factors is an important step, and IDH and IDHTN share some characteristics in common, such as age and comorbidities^9,10^. Nevertheless, known risk factors alone cannot successfully predict IDH and IDHTN when varying blood pressures (BPs) on hemodialysis are not considered^11–13^. Due to the repeated occurrence of IDH and IDHTN, information on previous hemodialysis sessions may help predict outcomes in the next session^12^. However, most studies used this information as binary features only and not as a concrete structure to encode all information^14,15^.

The dataset on hemodialysis sessions is complex due to its multiple variables with time series data. While some variables may be regularly arranged, others may not, and there may be missing data in certain areas of the sessions. The deep learning method has brought revolutionary advances in controlling the hemodialysis dataset because it successfully addresses time-series variables^9^. Previously, our recurrent neural network (RNN)-based model had favorable performance in predicting IDH^12^, although IDHTN was not considered, and the information from previous sessions was used as a handcrafted and binary feature. A recent deep learning model, named the temporal fusion transformer (TFT)^16^, efficiently encodes both time-invariant and time-varying variables after shrinking unimportant features. It has shown high performance with time-series forecasting tasks, such as stock index prediction and traffic occupancy rate prediction, and provides insights into which time step feature information is considered important^16^. The TFT-based model with a multihorizon forecasting feature yields predicted values at many future time steps, and is designed to consider both time-varying inputs (i.e., the future variable cannot know in the current time step) and time-invariant variables (i.e., the past observed variable and the known future variable we can expect) at a single inference. Nevertheless, the hemodialysis dataset contains many missing values, which makes it difficult to directly apply the original version of TFT to handle it. Continuous quantile prediction, used to conservatively estimate and predict outputs for the purpose of risk management in the original TFT, did not fit our task. Therefore, we modified the internal structure of TFT to impute the missing values by encoding all timestamps from previous sessions. We also adjusted the output types to enable real-time prediction of binary outcomes, achieving high performance in predicting both IDH and IDHTN using a hemodialysis dataset. Accordingly, the model performance outperformed that of other machine learning models, such as RNN, light gradient boosting machine (LGBM), random forest (RF), and logistic regression (LR). The explainable attributions for each case may provide clinicians with insights on how to manage the next session to reduce the risk of IDH and IDHTN.

## Methods

### Data source and study approval

A retrospective analysis of electrical medical record (EMR) data was performed. The data from 342,361 sessions of 12,200 patients who underwent hemodialysis at Seoul National University Hospital between October 2004 and December 2020 were retrieved from the EMR system and our own vital sign registry (named CONTINUAL)^17^. The institutional review board of Seoul National University Hospital approved the study design (no. H-2008-142-1151), and the study was conducted in accordance with the principles of the Declaration of Helsinki. Our study was retrospective analysis, and thus, getting informed consent from subjects was waived from the institutional review board of Seoul National University Hospital.

### Data collection and preparation

Sessions from patients who were < 18 years old (n = 20,014), had no initial BP records (n = 530), received < 2 h or > 6 h of hemodialysis (n = 9,647), and had > 1.5 h of time interval between BPs (n = 9396) were excluded. Accordingly, 302,774 sessions from 11,110 patients were ultimately used for model development. When there were no specific episodes or complications during hemodialysis, vital signs were monitored every hour. If vital signs became unstable, they were monitored more frequently.

**Figure 1:**
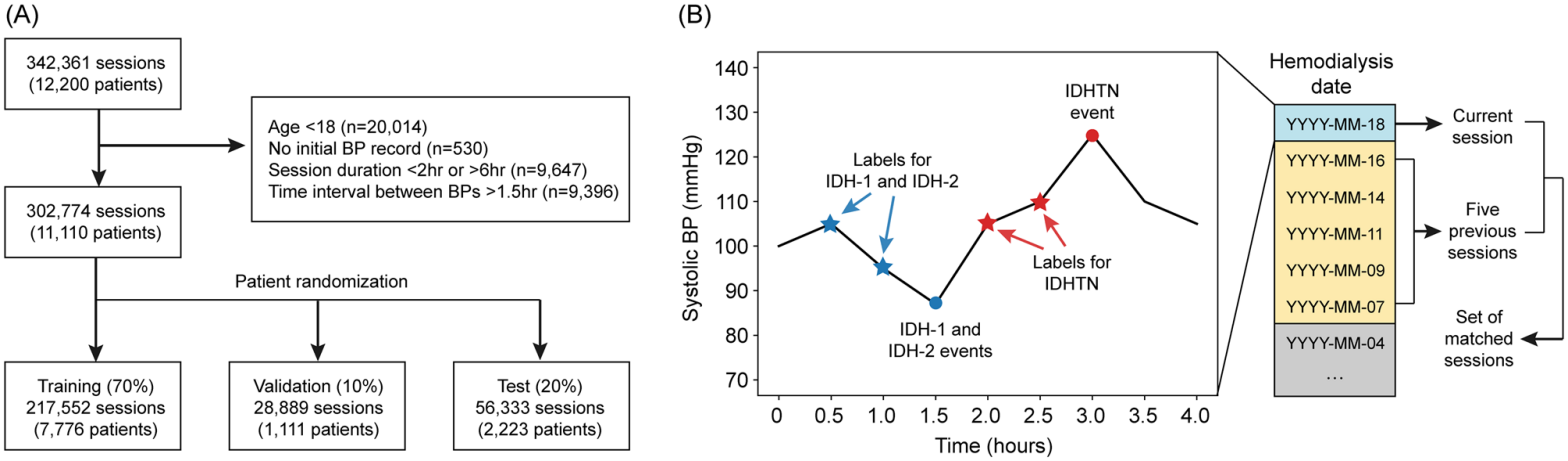
Dataset development. (**A**), Flow chart of data collection and randomization. (**B**), An illustrative example of labeling and creating a set of matched sessions.

The sessions were randomly split into training (70%), validation (10%), and test (20%) datasets based on patient information (Fig. 1A). The training and validation datasets were used for model development, while the test dataset was kept for evaluation. Each hemodialysis session was matched with up to 5 previous sessions within 1 month, and the set containing matched sessions was created for analysis (Fig. 1B). If dialysis information was not available for any of the previous 5 dialysis sessions within 1 month, the information on previous sessions was treated as missing values and ignored by attention masking.

The dataset used to train the model contained a total of 66 variables, such as baseline characteristics, hemodialysis setting, vital signs, laboratory findings, and medications used. The time-varying variables included vital signs and hemodialysis setting, while others were time-invariant. The list and relevant missing ratio are available in Supplementary Table 1. For time-invariant variables, missing values were imputed by means for continuous variables with normal distribution, or medians for both continuous variables without normal distribution and categorical variables. A forward-filling method was used for time-varying variables. To enable the model to recognize missing values, we created an auxiliary column that indicates whether imputation was performed at each time step for every time-varying variable. The auxiliary column was encoded with 0 if imputation was conducted (i.e., the variable was filled with information from the previous session) or 1 if the original observed value was used. After imputation of the missing values, continuous variables were normalized using the mean and the standard deviation. Accordingly, 78 variables with 66 original variables and 10 auxiliary variables indicating imputation and 2 variables of elapsed times (e.g., hemodialysis time and interval with the previous session) were used in a training dataset.

### Study outcomes

IDH was defined when systolic BP < 90 mmHg (termed IDH-1) and a decrease in systolic BP ≥ 20 mmHg and/or a decrease in mean arterial pressure ≥ 10 mmHg from the initial BP (termed IDH-2) occurred^18,19^. IDHTN was defined as an increase in systolic BP ≥ 10 mmHg from the initial systolic BP^20,21^. Because previous randomized controlled studies adopted the definition of IDHTN over multiple sessions^22–25^, we defined IDHTN-2 as cases with IDHTN occurring ≥ 4 of 6 consecutive sessions and applied it as a sensitivity analysis. Mean arterial pressure was calculated as ([2 × diastolic BP] + systolic BP) / 3. The occurrence of IDH and IDHTN within 1 h was labeled for model training (Fig. 1B).

### Model development

To predict IDH and IDHTN, time-varying and time-invariant variables were considered simultaneously, all of which were dealt with in the TFT-based model. The TFT architecture was originally proposed to address multihorizon forecasting problems in various domains^16^, and we employed the architecture and modified several components of the original model to fit our task. The model has been designed to consider both short- and long-term temporal relationships to produce predictions with sequence-to-sequence and attention mechanisms, respectively.

**Figure 2:**
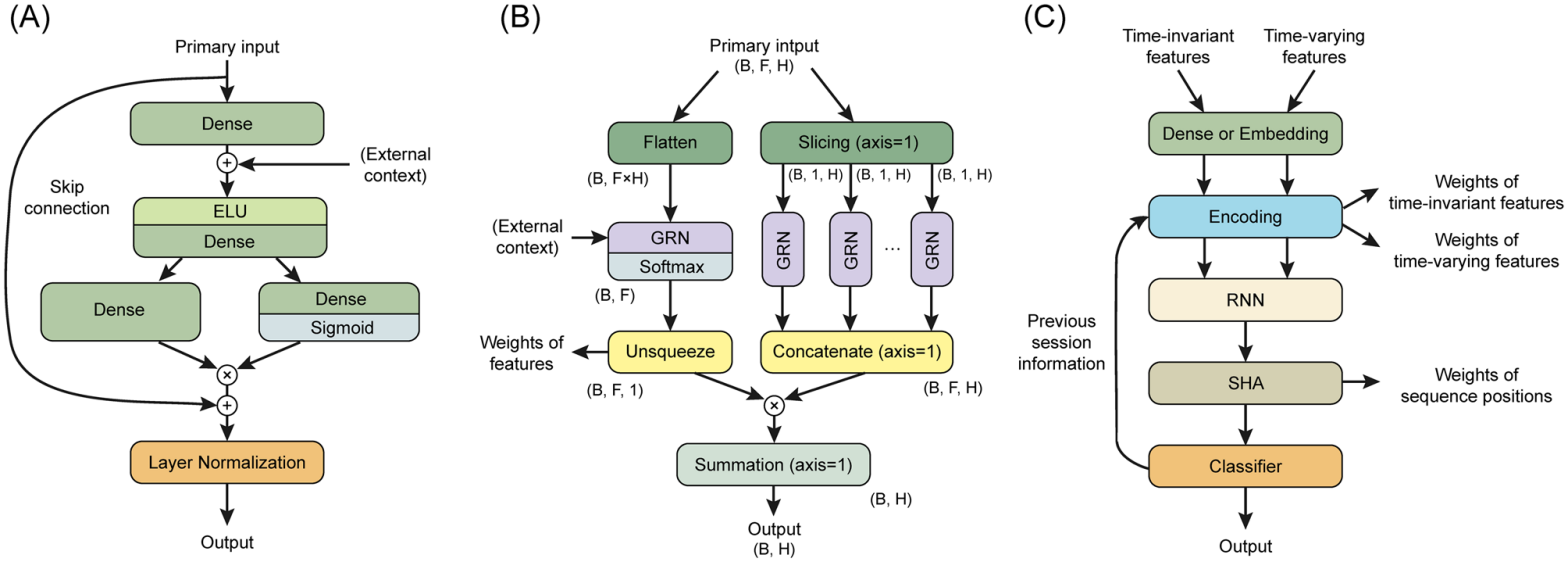
Schematic diagram of the model structure. (**A**), The internal structure of gated residual units (GRNs). (**B**), Encoding module with GRN. Encoded feature shapes are presented as (B, F, H) or (B, H). (**C**), Feature processing pipeline. B, batch size; F, feature shape; H, hidden shape. RNN, recurrent neural network; SHA, single-headed attention.

To address the challenge of determining the precise relationship between unforeseen exogenous input variables and targets, we employed the gated residual network (GRN) as the primary computing layer in the TFT architecture. It comprised four dense layers and two activation layers (i.e., an exponential linear unit function and a sigmoid function) (Fig. 2A), and determined whether to skip the features. Note that GRN is used in the encoding module (detailed in Fig. 2B), the input layer of the single-headed attention module (the fourth layer of Fig. 2C), and the input layer of the classifier (the last layer of Fig. 2C).

The TFT architecture could yield importance scores among time-varying and time-invariant variables and timestamps from the variable selection module and single-headed attention module, respectively. The variable selection module chose pertinent input variables at each time step, and we extracted the attention weight that explained the feature importance from the module. The higher attention weight indicates a higher contribution to predicting the output (see the second layer of Fig. 2C). Figure 2B represents the encoding flow of generating variable selection weights. The processed inputs were multiplied by this module, and unnecessary inputs at each time step could be removed. The single-headed attention module captured the long-term relationships within inputs by preserving the causality information flow across multiple time steps. We extracted the attention weight that explained the temporal importance of the timestamps (see the fourth layer of Fig. 2C). This operation is identical to the structure of the paper that was first proposed, and a detailed process can be found in that paper^26^.

Finally, Fig. 2C summarizes the entire pipeline of data processing of the TFT architecture. Time-invariant and time-varying inputs were separately transformed by dense or embedding layers for continuous and categorical inputs, respectively. The features from the first layer were sequentially processed by encoding, RNN, single-headed attention, and classifier modules. In particular, the RNN module analyzed the short-term relationship of inputs by leveraging local context using a gated recurrent unit^27^, and the classifier module produced output by composing features from the previous layer with a single dense layer. Information on previous sessions was re-entered into the encoding module as time-invariant features of the current session after passing the pipeline. If there was no available information on previous sessions, the feature weight of the corresponding session in the encoding module was set to zero and did not affect the downstream calculation. The model finally calculated the probabilities of IDH-1, IDH-2, and IDHTN as outcomes. The detailed structure and source codes in Python are provided in https://github.com/dactylogram/HD_IDH_prediction/.

Other machine learning models, such as RNN, Light Gradient Boosting Machine, Random forest, and logistic regression were compared with the TFT-based model in predicting outcomes. The RNN model had the same structure as that in a previous study^12^, and it could handle time-varying features but did not use the information from the previous session. Other models could handle tabular datasets alone, and three timestamps, such as initiation, prediction, and the previous entry. The detailed methods and designs of canonical machine learning and deep learning models are presented in the Supplementary Methods.

### Model evaluation

The area under the receiver operating characteristic curve (AUROC) and the area under the precision-recall curve (AUPRC) were used to evaluate the model performance. Comparisons between AUROCs was calculated by the DeLong test. Calibration ability was applied using a calibration plot. Evaluation metrics of precision, recall, and F1 score were also evaluated. All evaluation metrics were obtained from the test dataset. We set a threshold of 0.5 to determine the binary outcomes. The formulae with true positive (TP), false positive (FP), and false negative (FN) counts are represented as follows.

$$\mathrm{Precision}= \frac{\mathrm{TP}}{\mathrm{TP}+\mathrm{FP}}$$

$$\mathrm{Recall}= \frac{\mathrm{TP}}{\mathrm{TP}+\mathrm{FN}}$$

$$\mathrm{F}1\mathrm{ score}=2\times \frac{\mathrm{Precision}\times \mathrm{Recall}}{\mathrm{Precision}+\mathrm{Recall}}$$

## Results

### Baseline characteristics

The baseline characteristics of clinical information and the hemodialysis sessions are presented in Table 1. Statistics for the variables in the dataset were described for each session. The mean age of the patients across the sessions was 62 ± 15 years, and 57.7% were female. The prevalence rates of diabetes mellitus and hypertension were 48.7% and 72.9%, respectively. The median values of initial systolic and diastolic BPs were 139 mmHg and 73 mmHg, respectively. The number of BP recordings per session was 7.1 ± 3.5.

**Table 1:** Baseline characteristics of the hemodialysis sessions.

Variables	Total (n = 302,774)
Age (years)	62.1 ± 15.2
Female (%)	57.7
Hemodialysis type (%)	
Hemodialysis	92.6
Online hemodiafiltration	7.0
Ultrafiltration	0.2
Others	0.2
Vascular access (%)	
Arteriovenous fistula	71.5
Subcutaneously tunneled catheter	17.5
Nontunneled venous catheter	5.7
Arteriovenous graft	5.3
Predialytic weight (kg)	59.5 ± 12.2
Initial blood flow rate (ml/min)	250 (230–280)
Initial systolic blood pressure (mmHg)	139 (121–155)
Initial diastolic blood pressure (mmHg)	73 (65–83)
Initial heart rate (/min)	75 (66–86)
Initial respiratory rate (/min)	18 (16–18)
Initial body temperature (°C)	36.3 (36.1–36.6)
Blood findings	
Hemoglobin (g/dL)	10.4 (9.4–11.2)
Albumin (g/dL)	3.7 (3.2–4.0)
Calcium (mg/dL)	8.9 (8.3–9.4)
Phosphate (mg/dL)	4.4 (3.4–5.4)
Sodium (mmol/L)	137 (135–139)
Potassium (mmol/L)	4.6 (4.1–5.2)
Comorbidities (%)	
Diabetes mellitus	48.7
Hypertension	72.9
Coronary artery disease	24.6
Medications (%)	
Antihypertensive drugs	72.2
Oral hypoglycemic agents	11.7
Subcutaneous insulin	25.6
Statins	35.5

IDH occurred in 54.1% (IDH-1 in 10.7%, and IDH-2 in 51.9%), and IDHTN occurred in 40.5%. IDHTN-2 definition was available in 95.5% of hemodialysis sessions, and the prevalence of IDHTN-2 was 23.1%. The occurrences of IDH-1, IDH-2 and IDHTN as the dialysis progressed were present in Supplementary Fig. 1. While the occurrence of IDH-1, IDH-2 and IDHTN did not largely change as the dialysis progressed, we observed an increase in IDH-1, IDH-2 and a decrease in IDHTN after nearly 4 h of dialysis. Detailed statistics from the training, validation, and test datasets are available in Supplementary Table 1.

### Model performance

**Figure 3:**
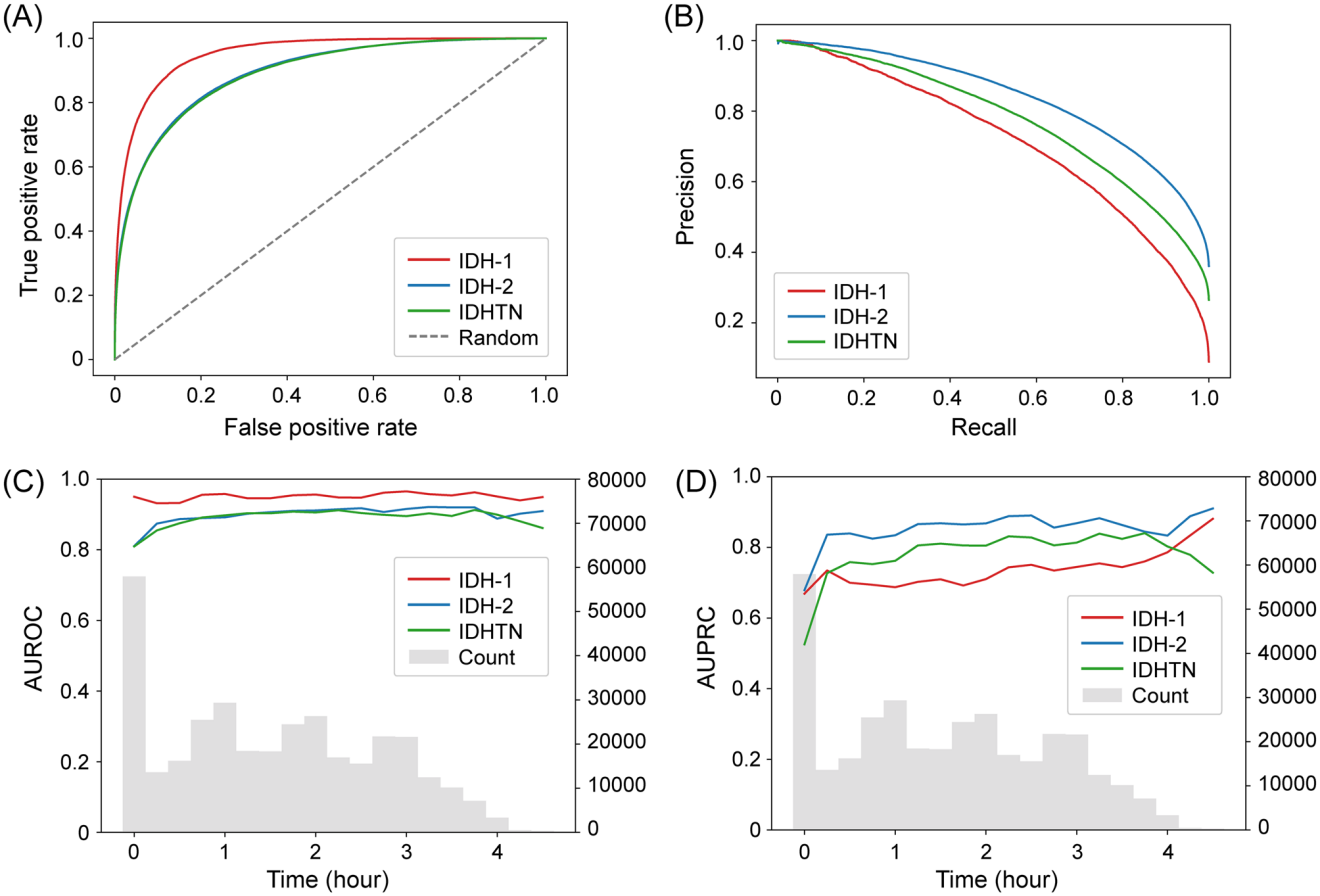
Plots of the model performance. (**A**), Area under the receiver operating curve (AUROC) in predicting outcomes. (**B**), Area under the precision-recall curve (AUPRC) in predicting outcomes. (**C**), AUROC of outcomes and timestamp count histogram according to the elapsed time. (**D**), AUPRC of outcomes and timestamp count histogram according to the elapsed time.

Table 2 represents AUROCs and AUPRCs as model performance. The TFT-based model achieved the highest AUROC value among the models evaluated (*P*s < 0.001), and the AUROCs for IDH-1, IDH-2, and IDHTN were 0.953 (0.952–0.954), 0.892 (0.891–0.893), and 0.889 (0.888–0.890), respectively (Fig. 3A). The values of AUPRCs for IDH-1, IDH-2, and IDHTN in the TFT-based model were also higher than those obtained from other machine learning models (Fig. 3B). The TFT-based model achieved the highest F1 scores compared to the other machine learning models in predicting IDH-1 (0.630), IDH-2 (0.738), and IDHTN (0.668, Supplementary Table 2). The TFT-based model was well calibrated (Supplementary Fig. 2).

**Table 2:** Model performance for predicting intradialytic hypotension and hypertension.

Outcomes	Models	AUROC (95% CI)	*P*	AUPRC (95% CI)
IDH-1	TFT-based	0.953 (0.952–0.954)	Reference	0.716 (0.714–0.717)
	RNN	0.929 (0.928–0.930)	< 0.001	0.624 (0.622–0.626)
	LightGBM	0.928 (0.926–0.929)	< 0.001	0.633 (0.631–0.635)
	Random forest	0.922 (0.921–0.924)	< 0.001	0.620 (0.618–0.621)
	Logistic regression	0.913 (0.911–0.915)	< 0.001	0.583 (0.582–0.585)
IDH-2	TFT-based	0.892 (0.891–0.893)	Reference	0.838 (0.836–0.839)
	RNN	0.864 (0.863–0.865)	< 0.001	0.803 (0.801–0.804)
	LightGBM	0.862 (0.861–0.863)	< 0.001	0.799 (0.797–0.800)
	Random forest	0.838 (0.836–0.839)	< 0.001	0.765 (0.763–0.766)
	Logistic regression	0.848 (0.847–0.849)	< 0.001	0.782 (0.780–0.783)
IDHTN	TFT-based	0.889 (0.888–0.890)	Reference	0.774 (0.773–0.776)
	RNN	0.868 (0.866–0.869)	< 0.001	0.745 (0.743–0.746)
	LightGBM	0.862 (0.861–0.864)	< 0.001	0.734 (0.732–0.735)
	Random forest	0.831 (0.830–0.833)	< 0.001	0.682 (0.681–0.684)
	Logistic regression	0.847 (0.846–0.849)	< 0.001	0.714 (0.713–0.716)

IDH-1, nadir systolic blood pressure < 90 mmHg; IDH-2, a decrease in systolic blood pressure ≥ 20 mmHg and/or a decrease in mean arterial pressure ≥ 10 mmHg; IDHTN, an increase in systolic blood pressure ≥ 10 mmHg.

AUROC, area under the receiver operating characteristic curve; CI, confidence interval; AUPRC, area under the precision-recall curve; TFT, Temporal Fusion Transformer; RNN, recurrent neural network; LightGBM, Light Gradient Boosting Machine.

The values of AUROCs and AUPRCs remained consistent over the course of hemodialysis time (Fig. 3C,D), except for the AUPRC values at the beginning and end of the period. The AUPRCs of all three labels were relatively low at the start, and the predictive performance for IDH-1 and IDH-2 improved after approximately 3.5 h, while the predictive performance for IDHTN decreased. These changes in AUPRC appeared to correlate with the label prevalence over the duration of hemodialysis, as described in Supplementary Fig. 1.

When predicting IDHTN-2, the predictability of the model for IDH-1 and IDH-2 remained consistent, while there was an increase in AUROC (from 0.889 to 0.917) and a decrease in AUPRC (from 0.774 to 0.728). The changes in evaluation metrics might be associated with the change in prevalence.

### Explainable feature importance

**Figure 4:**
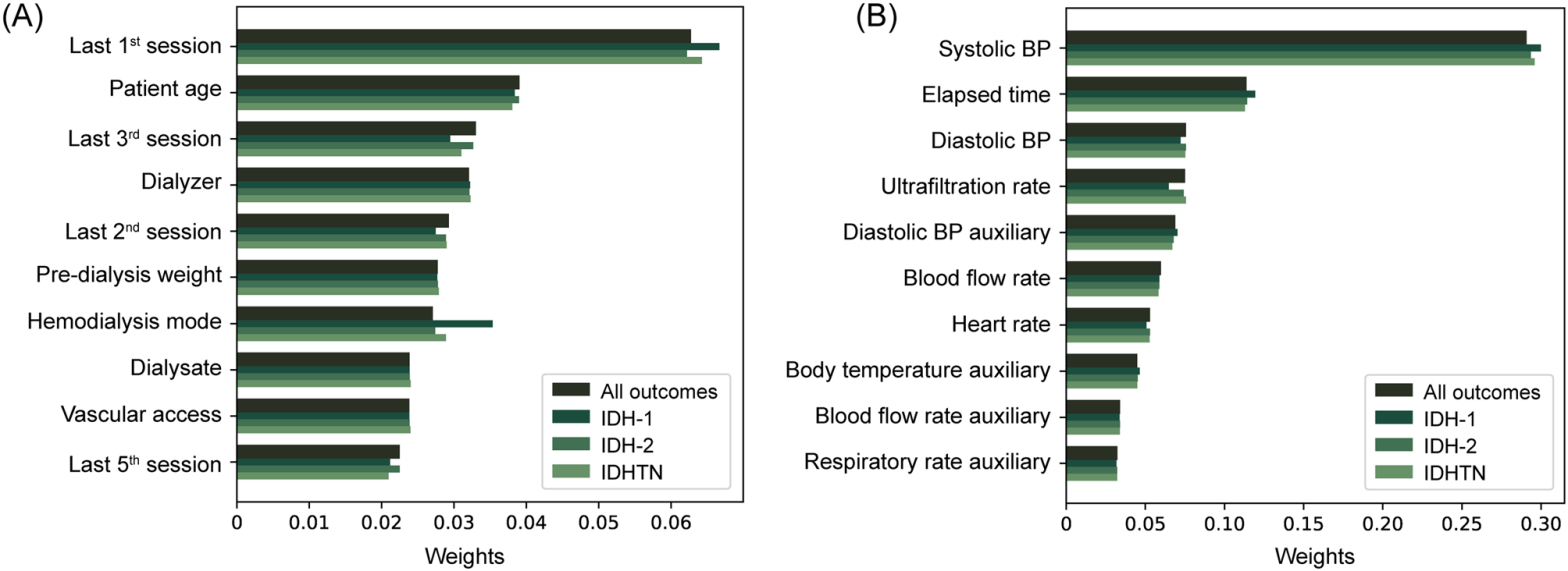
Mean weights of time-invariant and time-varying features from the attention module in the model. (**A**), Weights of time-invariant features. (**B**), Weights of time-varying features. BP, blood pressure.

The TFT-based model provides attention weights of features that indicate which is the most important part of the data to which the model paid attention, and the sum of attention weights is equal to one. The information of previous sessions, age, dialyzer type, and predialytic weight showed higher attention weights among time-invariant variables (Fig. 4A). Among the time-varying variables, systolic BP was highly ranked, and elapsed time, diastolic BP, and ultrafiltration rate were the most important variables for the prediction of IDH and IDHTN (Fig. 4B).

**Figure 5:**
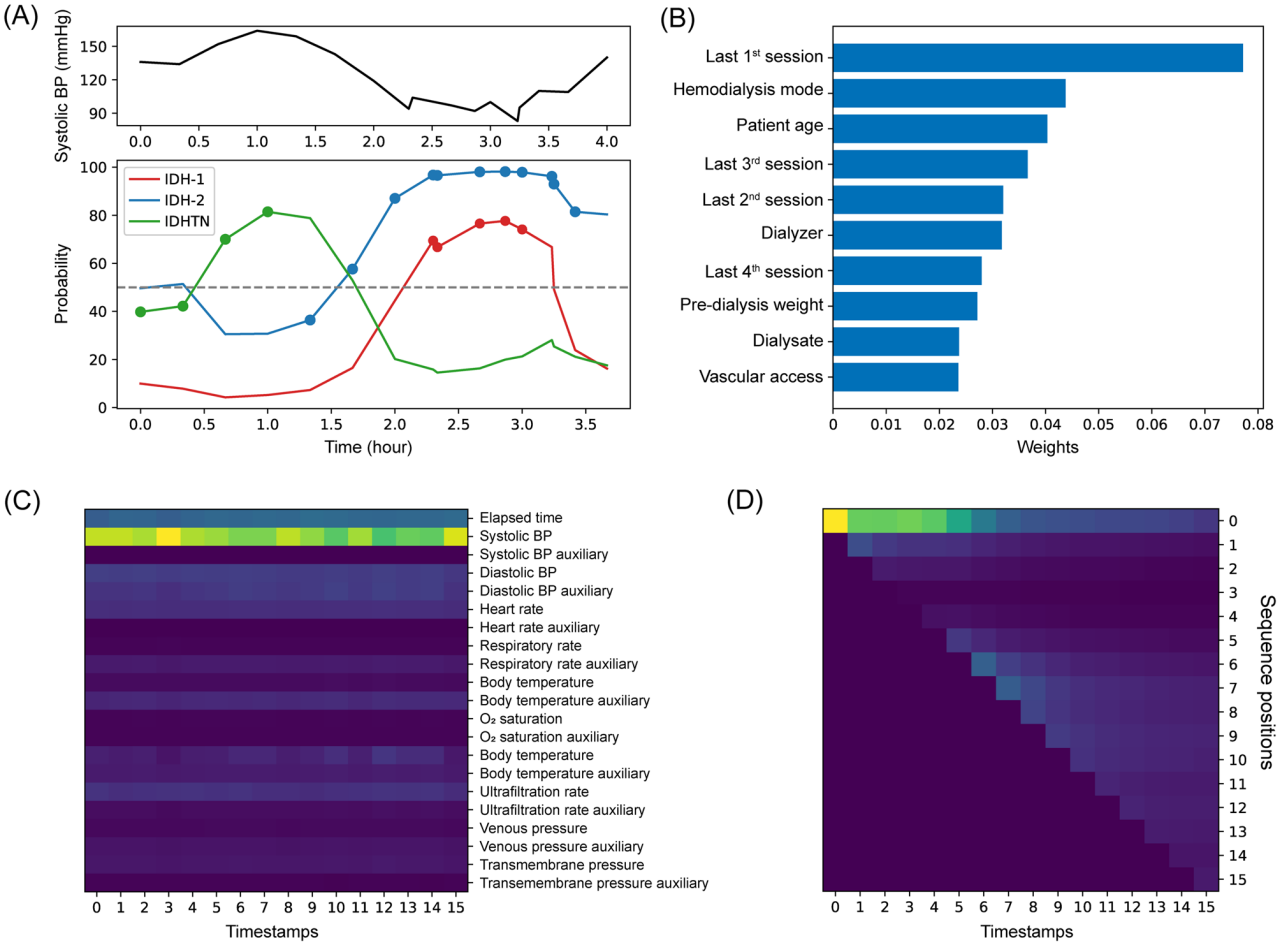
Explainability of one case with intradialytic hypotension (IDH) and hypertension (IDHTN). (**A**), Real-time prediction of outcomes using the model. Round circles on the lines of a probability represent true label positions. (**B**) Weights of time-invariant features by the model explainability. (**C**) Weights of time-varying features by the model explainability. (**D**) Weights of the sequence from single-headed attention according to the timestamps. The positions of the future sequences were masked, and their values were set to 0.

We provide an example of a session in which the presented TFT-based model can offer real-time probabilities for all outcomes (Fig. 5A). The occurrence of IDH and IDHTN could be explained by the attention weights of time-invariant and time-varying variables (Fig. 5B,C). Weights of time-varying variables from the encoding module and sequence positions from the single-headed attention module had slightly different values according to the timestamps (Fig. 5C,D), and systolic BP of the time-varying variable and the first timestamp (i.e., the initial recording of hemodialysis) of the single-headed attention sequence were highly related to the performance.

### Slim version of the TFT model

According to the variable attention weights, we selected the top 10 (i.e., 2 time-varying and 8 time-invariant) or 30 (i.e., 6 time-varying and 24 time-invariant) variables to develop the slim TFT-based model. The slim TFT-based model achieved acceptable performance compared to the parent model; particularly, the slim model with 30 variables had noninferior AUROCs to the parent model (Table 3). The AUPRCs were also similar between the slim model and the parent models (Table 3). The results indicate that the single-headed attention module of the TFT-based model efficiently prioritized the most crucial parts of the input data while disregarding irrelevant parts, effectively ranking variables in a significant order.

**Table 3:** Performance in the slim version of the model.

		Model with 10 variables		Model with 30 variables	
Outcomes	Performance	Matric value (CI)	P*	Matric value (CI)	P*
IDH-1	AUROC	0.948 (0.947–0.950)	< 0.001	0.952 (0.951–0.953)	0.376
	AUPRC	0.699 (0.698–0.701)	NA	0.712 (0.711–0.714)	NA
IDH-2	AUROC	0.889 (0.888–0.890)	0.002	0.891 (0.889–0.892)	0.128
	AUPRC	0.834 (0.833–0.835)	NA	0.836 (0.835–0.837)	NA
IDHTN	AUROC	0.888 (0.887–0.889)	0.153	0.890 (0.888–0.891)	0.478
	AUPRC	0.773 (0.772–0.775)	NA	0.777 (0.772–0.775)	NA

IDH-1, nadir systolic blood pressure < 90 mmHg; IDH-2, a decrease in systolic blood pressure ≥ 20 mmHg and/or a decrease in mean arterial pressure ≥ 10 mmHg; IDHTN, an increase in systolic blood pressure ≥ 10 mmHg.

CI, confidence interval; AUROC, area under the receiver operating characteristic curve; AUPRC, area under the precision-recall curve; NA, not applicable.

*P values compared to the parent model using all variables.

### Implication of the previous session in the model performance

**Figure 6:**
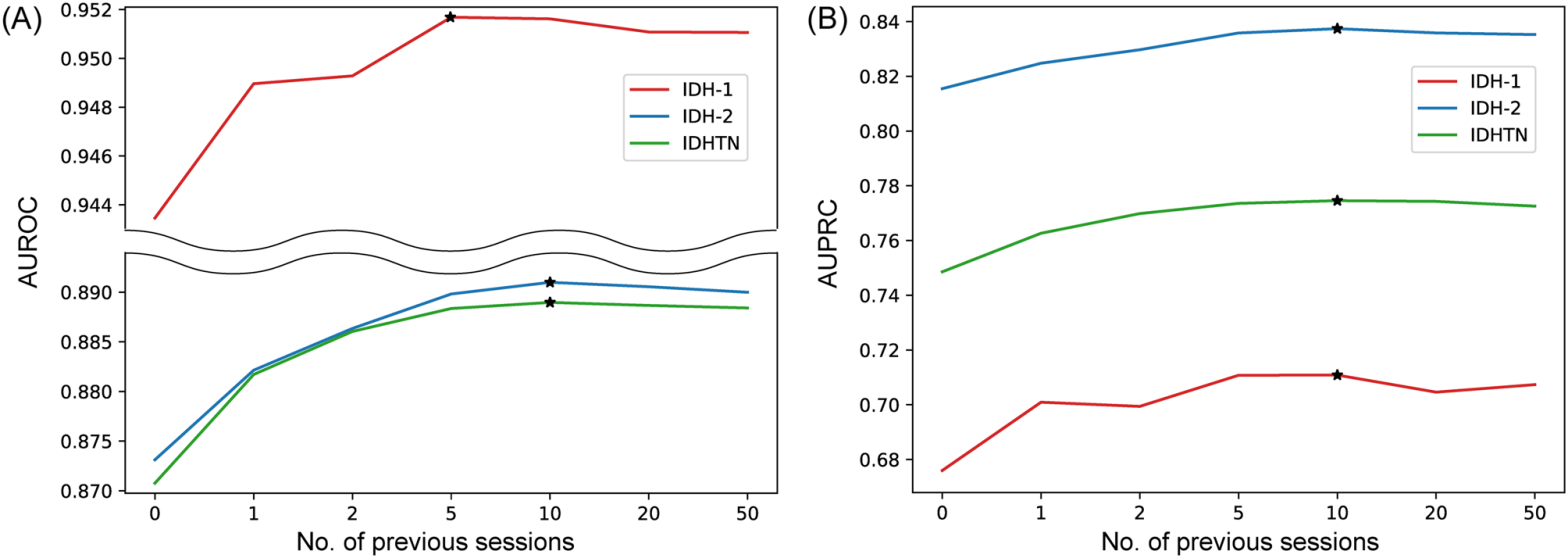
Changes in AUROC (**A**) and AUPRC (**B**) according to the number of previous sessions used in the slim version model. *Maximum point of the performance metrics.

To determine whether adding information about previous sessions to the model improves performance, AUROCs and AUPRCs were calculated depending on the number of previous sessions used in the model. Herein, the slim TFT-based model was used because of our limit in GPU memory. Both the AUROC and AUPRC achieved maximum values when 5 to 10 previous sessions were used in the model (Fig. 6). The model performance did not improve significantly even with > 10 previous sessions, implying that information on the most recent previous session was more valuable for prediction than data from the remote sessions.

## Discussion

The present TFT-based model could encode information from previous sessions as a time-invariant variable and achieve state-of-the-art performance in predicting both IDH and IDHTN in real-time. Another strength of the model was its ability to provide explainable variable importance and timestamps through attention weights. Model explainability could be utilized to reduce the number of required input variables without compromising performance. Information from previous sessions significantly contributed to predicting outcomes, with data from recent sessions proving more valuable than remote sessions in enhancing model performance.

Deep learning has been applied to predict the binary occurrence of intradialytic complications, such as IDH, IDHTN, and other clinical symptoms, using a tabular dataset of hemodialysis sessions^14,15,28,29^. We developed the RNN model to predict IDH in real-time using a time-series dataset, and its performance was acceptable^12^. In this study, to further enhance performance, we employed the TFT architecture with specific modifications, including handling missing values and implementing binary classification methods. As a result, our performance exceeded that of other machine learning or deep learning models. Although the number of input variables used has decreased, the slim TFT-based model still outperformed other machine learning or deep learning models in prediction outcomes. The results are attributable to the internal characteristics of the TFT architecture, such as effective feature extraction by removing unimportant features using GRN and encoding modules.

There have been several studies that utilized the hand-crafted (e.g., the lowest systolic BP) or selected (e.g., pre- or post-dialysis BP) information on the previous sessions to predict events in the subsequent session^30,31^. To the best of our knowledge, the present study represents the first attempt to incorporate entire sequences of previous hemodialysis sessions. The information on previous sessions could be encoded and integrated as time-invariant features in the model training. This process amplified the prediction performance, although a plateau in improvement was observed after the number of previous sessions exceeded 10. The results suggest that information on the recent previous session was more useful in prediction than that on the remote session, and a proper number of previous sessions was sufficient to achieve good performance, which would lead to less computational cost.

The present TFT-based model could provide information on feature attention weights, which we named explainability. There are indirect post-hoc methods to rank features, such as SHapley Additive exPlanations^32^ and class activation maps^33^, whereas the TFT-based model can directly correlate the rank of features with the internal single-headed attention module. The slim TFT-based model showed similar performance to the parent model only with subset variables because the importance ranking of features produced by the model was reliable.

Although the study results are informative, there are certain limitations to be discussed. Session information was mainly derived from patients with end-stage kidney disease; thus, our model performance may differ in the cases involving acute kidney injury requiring hemodialysis. The model primarily emphasized BP-related outcomes and did not address other intradialytic complications, such as arrhythmia, high pulse pressure, and sudden death. Certain time-invariant or time-varying variables (e.g., dialysis vintage and electrocardiogram) would be additionally helpful to predict IDH or IDHTN.

## Conclusion

The present study introduces a novel model for real-time simultaneous prediction of IDH and IDHTN by incorporating information from previous sessions. Furthermore, the model provides feature attention weights to elucidate the significance of variables in the context of IDH or IDHTN. Accordingly, this explainable model assists clinicians in preparing for IDH and IDHTN in advance. The results of this study may also serve as an inspiration for other researchers to leverage information from previous sessions to improve the model performance in predicting intradialytic complications.

### Supplementary Information

Supplementary Information.

## Data Availability

Python code for model structure and describing dataset structures are provided in https://github.com/dactylogram/HD_IDH_prediction/. The other data including model training are available from the corresponding author upon request.
