# Supplementary material for: Real-time dual prediction of intradialytic hypotension and hypertension using an explainable deep learning model
Source: Sci Rep. 2023 Oct 23;13:18054. doi: 10.1038/s41598-023-45282-1 (PMC10593747; doi:10.1038/s41598-023-45282-1)

**Supplementary materials**

Supplementary Methods.

Supplementary Table 1. Baseline characteristics of study variables according to dataset.

Supplementary Table 2. Precision, recall, and F1 scores for predicting intradialytic hypotension and hypertension.

Supplementary Table 3. Model performance for predicting intradialytic hypotension and extendedly defined intradialytic hypertension.

Supplementary Figure 1. Timestamp count histogram and the occurrence of IDH-1, IDH-2, and IDHTN according to dialysis time.

Supplementary Figure 2. Calibration plot of the TFT-based model.

**Supplementary Methods**

**Development of the recurrent neural network model**

Python (version 3.9.7) and PyTorch (version 1.8.2) were used to develop the recurrent neural network model. After being transformed by batch normalization, features were passed by one linear layer, two stacked GRU layers, and three linear layers. The sigmoid activation function was followed by the last linear layer, while ReLU activation was followed by the other linear layers. Layer normalization and dropout layers were present after the second and third linear layers, respectively. The binary cross-entropy loss and the Adam optimizer were used in the model training.

**Development of other machine learning models**

Python (version 3.9.7) and Scikit-Learn (version 1.0.2) were used to develop light gradient boosting machine, random forest, and logistic regression models. Hyperparameter tuning was performed based on the performance in the validation set, and the hyperparameters are listed below.

| Models | Hyperparameters |
| --- | --- |
| Light gradient boosting machine | n_estimators (100, 200, 400)  learning_rate (0.1, 0.2)  num_leaves (31, 62) |
| Random forest | criterion (Gini, Entropy)  n_estimators (100, 200, 300)  max_depth (3, 6, 12) |
| Logistic regression | penalty (L1, L2)  C (0.01, 0.1, 1, 10) |

**Supplementary Table 1. Baseline characteristics of study variables according to dataset.**

| **Variables** | | | | | **Total** | **Train set** | **Validation set** | **Test set** |
| --- | --- | --- | --- | --- | --- | --- | --- | --- |
| **Name** | | **Type** | **Category** | **Missing**  **ratio (%)** | **Statistics*** | | | |
| Demographics | Patient age (years) | Time-invariant | - | 0 | 62.1 +- 15.2 | 62.1 +- 14.5 | 63.0 +- 16.1 | 61.4 +- 17.1 |
|  | Female (%) | Time-invariant | - | 0 | 57.7 | 57.3 | 65.6 | 54.9 |
| Hemodialysis settings | Hemodialysis type (%) | Time-invariant | Hemodialysis | 0 | 92.6 | 91.7 | 93.9 | 95.4 |
|  |  |  | Hemodiafiltration |  | 7.0 | 7.8 | 5.9 | 4.2 |
|  |  |  | Ultrafiltration |  | 0.2 | 0.2 | <0.1 | 0.2 |
|  |  |  | Hemoperfusion |  | <0.1 | 0.1 | <0.1 | <0.1 |
|  |  |  | Hemodialysis with sorbent regeneration of dialysate |  | <0.1 | <0.1 | <0.1 | <0.1 |
|  | Vascular access (%) | Time-invariant | Arteriovenous fistula | 0 | 71.5 | 71.7 | 72.2 | 70.3 |
|  |  |  | Subcutaneously-tunneled catheter |  | 17.5 | 16.7 | 17.2 | 20.8 |
|  |  |  | Temporary catheter |  | 5.7 | 5.9 | 6.0 | 5.6 |
|  |  |  | Arteriovenous graft |  | 5.3 | 5.7 | 4.6 | 3.4 |
|  | Pre-dialysis weight (kg) | Time-invariant | - | <0.1 | 59.5 +- 12.2 | 59.9 +- 11.5 | 57.8 +- 12.9 | 59.1 +- 14.1 |
|  | Dialyzer surface (m^2) | Time-invariant | - | 0 | 1.4 (1.4 - 1.4) | 1.4 (1.4 - 1.4) | 1.4 (1.4 - 1.4) | 1.4 (1.4 - 1.4) |
|  | Priming fluid (%) | Time-invariant | Saline | 0 | 79.2 | 80.1 | 76.8 | 77.3 |
|  |  |  | None |  | 17.2 | 16.5 | 18.9 | 19.0 |
|  |  |  | Half |  | 3.5 | 3.4 | 4.2 | 3.7 |
|  |  |  | Blood |  | <0.1 | <0.1 | <0.1 | <0.1 |
|  | Dialysate (%) | Time-invariant | B dex 0.1% | <0.1 | 78.7 | 79.3 | 79.3 | 76.1 |
|  |  |  | B dex 0.15% |  | 15.8 | 15.2 | 14.0 | 19.0 |
|  |  |  | B 1/5L |  | 5.5 | 5.6 | 6.7 | 4.9 |
|  | Dialyzer machine (%) | Time-invariant | polyflux 14L | 0 | 54.4 | 53.9 | 55.4 | 55.5 |
|  |  |  | polyflux 170H |  | 10.0 | 11.2 | 12.3 | 9.5 |
|  |  |  | FX 5 |  | 9.5 | 9.1 | 6.4 | 7.5 |
|  |  |  | F5 HPS |  | 5.8 | 6.0 | 5.0 | 5.6 |
|  |  |  | Theranova 400 |  | 5.3 | 5.2 | 4.9 | 5.5 |
|  |  |  | APS-15U |  | 4.9 | 4.9 | 4.2 | 4.9 |
|  |  |  | FX 50 |  | 2.5 | 2.3 | 3.7 | 2.5 |
|  |  |  | Revaclear 300 |  | 2.2 | 2.2 | 2.3 | 2.4 |
|  |  |  | Rexeed-15A |  | 1.3 | 1.3 | 1.5 | 1.7 |
|  |  |  | polyflux 17S |  | 0.9 | 1.0 | 1.2 | 1.3 |
|  |  |  | Xevonta Hi 15 |  | 0.4 | 0.5 | 0.8 | 0.6 |
|  |  |  | BLS 812 G |  | 0.4 | 0.4 | 0.5 | 0.5 |
|  |  |  | BLS 819 SD |  | 0.4 | 0.3 | 0.4 | 0.5 |
|  |  |  | FX 40 |  | 0.4 | 0.3 | 0.4 | 0.4 |
|  |  |  | polyflux 140H |  | 0.2 | 0.2 | 0.3 | 0.3 |
|  |  |  | F4 HPS |  | 0.2 | 0.2 | 0.1 | 0.2 |
|  |  |  | Revaclear 400 |  | 0.2 | 0.2 | 0.1 | 0.2 |
|  |  |  | polyflux 14S |  | 0.2 | 0.2 | 0.1 | 0.2 |
|  |  |  | SG 30 |  | 0.2 | 0.1 | 0.1 | 0.2 |
|  |  |  | polyflux 8L |  | 0.1 | 0.1 | <0.1 | 0.1 |
|  |  |  | polyflux 6H |  | 0.1 | 0.1 | <0.1 | 0.1 |
|  |  |  | BLS 816 SD |  | 0.1 | 0.1 | <0.1 | 0.1 |
|  |  |  | F6 HPS |  | 0.1 | 0.1 | <0.1 | 0.1 |
|  |  |  | NC 1485 |  | 0.1 | <0.1 | <0.1 | <0.1 |
|  |  |  | Others |  | 0.1 | 0.1 | <0.1 | <0.1 |
|  | Dialyzer membrane (%) | Time-invariant | Low | 0 | 70.4 | 69.7 | 73.2 | 71.3 |
|  |  |  | High |  | 24.4 | 25.0 | 20.4 | 23.7 |
|  |  |  | Mid |  | 5.3 | 5.2 | 6.4 | 4.9 |
|  |  |  | None |  | <0.1 | <0.1 | <0.1 | <0.1 |
|  | Dialysate flow rate (mL/min) | Time-invariant | - | 0 | 500 (500 - 500) | 500 (500 - 500) | 500 (500 - 500) | 500 (500 - 500) |
| Vital signs | Systolic BP (mmHg) | Time-varying | - | 0 | 139 (122 - 155) | 139 (121 - 154) | 138 (119 - 154) | 140 (125 - 156) |
|  | Diastolic BP (mmHg) | Time-varying | - | 0 | 73 (65 - 83) | 73 (64 - 82) | 74 (65 - 83) | 75 (66 - 85) |
|  | Heart rate (mmHg) | Time-varying | - | 0.3 | 76 (67 - 86) | 75 (66 - 86) | 76 (67 - 86) | 78 (68 - 88) |
|  | Respiratory rate (/min) | Time-varying | - | 15.8 | 18 (16 - 18) | 18 (16 - 18) | 18 (16 - 18) | 18 (16 - 18) |
|  | Body temperature (°C) | Time-varying | - | 0.4 | 36.3 (36.1 - 36.6) | 36.3 (36.1 - 36.6) | 36.3 (36.1 - 36.6) | 36.4 (36.1 - 36.6) |
|  | SpO2 (%) | Time-varying | - | 87.8 | 99 (97 - 100) | 99 (97 - 100) | 99 (97 - 100) | 99 (97 - 100) |
| Hemodialysis machine information | Blood flow rate (mL/min) | Time-varying | - | <0.1 | 250 (230 - 280) | 250 (230 - 280) | 250 (200 - 280) | 250 (220 - 280) |
|  | Ultrafiltration (kg) | Time-varying | - | 0.4 | 1.8 (0.9 - 2.6) | 1.8 (1.0 - 2.6) | 1.8 (0.8 - 2.6) | 1.8 (0.9 - 2.6) |
|  | Venous pressure (mmHg) | Time-varying | - | 0.2 | 106.0 (75.0 - 136.0) | 108.0 (76.0 - 137.0) | 106.0 (75.0 - 136.0) | 100.0 (70.0 - 130.0) |
|  | Transmembrane pressure (mmHg) | Time-varying | - | 29.4 | 32.0 (11.0 - 55.0) | 32.0 (12.0 - 55.0) | 31.0 (11.0 - 53.0) | 30.0 (10.0 - 54.0) |
| Clinical information | Diabetes mellitus (%) | Time-invariant | - | 0 | 48.7 | 50.4 | 46.2 | 43.6 |
|  | Hypertension (%) | Time-invariant | - | 0 | 72.9 | 71.6 | 73.4 | 77.8 |
|  | Coronary artery disease (%) | Time-invariant | - | 0 | 24.6 | 26.6 | 18.0 | 20.1 |
|  | Atrial fibrillation (%) | Time-invariant | - | 0 | 8.7 | 9.3 | 7.6 | 6.7 |
|  | Liver cirrhosis (%) | Time-invariant | - | 0 | 6.4 | 7.0 | 4.9 | 4.7 |
|  | Previous history of glomerulonephritis (%) | Time-invariant | - | 0 | 14.5 | 14.0 | 15.5 | 16.1 |
|  | Cancer (%) | Time-invariant | - | 0 | 24.8 | 25.7 | 20.8 | 23.6 |
|  | Kidney transplanation donor (%) | Time-invariant | - | 0 | <0.1 | <0.1 | <0.1 | <0.1 |
|  | Kidney transplanation recipient (%) | Time-invariant | - | 0 | 7.9 | 8.8 | 6.5 | 5.3 |
| Laboratory findings | White blood cell (10^9/L) | Time-invariant | - | 3.8 | 6.2 (4.8 - 8.2) | 6.3 (4.9 - 8.2) | 5.9 (4.7 - 7.7) | 6.2 (4.7 - 8.2) |
|  | Hemoglobin (g/dL) | Time-invariant | - | 3.7 | 10.4 (9.4 - 11.2) | 10.4 (9.5 - 11.2) | 10.4 (9.4 - 11.2) | 10.3 (9.4 - 11.1) |
|  | Hematocrit (%) | Time-invariant | - | 3.7 | 31.7 (28.9 - 34.2) | 31.8 (29.0 - 34.3) | 31.6 (28.8 - 34.2) | 31.4 (28.6 - 33.9) |
|  | Platelet (10^9/L) | Time-invariant | - | 3.8 | 168 (122 - 219) | 166 (121 - 218) | 155 (112 - 209) | 180 (134 - 226) |
|  | Cholesterol (mg/dL) | Time-invariant | - | 13.8 | 141 (118 - 167) | 142 (118 - 168) | 138 (118 - 164) | 139 (118 - 162) |
|  | Protein (g/dL) | Time-invariant | - | 5.0 | 6.6 (6.1 - 7.0) | 6.6 (6.1 - 7.0) | 6.6 (6.0 - 7.0) | 6.6 (6.0 - 7.0) |
|  | Albumin (g/dL) | Time-invariant | - | 5.1 | 3.7 (3.2 - 4.0) | 3.7 (3.2 - 4.0) | 3.6 (3.2 - 4.0) | 3.6 (3.1 - 4.0) |
|  | Total bilirubin (mg/dL) | Time-invariant | - | 5.1 | 0.4 (0.3 - 0.6) | 0.4 (0.3 - 0.6) | 0.4 (0.3 - 0.6) | 0.4 (0.3 - 0.6) |
|  | AST (IU/L) | Time-invariant | - | 5.1 | 18 (13 - 25) | 18 (13 - 25) | 18 (14 - 26) | 18 (13 - 26) |
|  | ALT (IU/L) | Time-invariant | - | 5.1 | 13 (9 - 20) | 13 (9 - 20) | 14 (10 - 21) | 12 (9 - 19) |
|  | ALP (IU/L) | Time-invariant | - | 5.1 | 81 (60 - 113) | 80 (60 - 111) | 78 (59 - 109) | 87 (62 - 121) |
|  | Prothrombin time (sec) | Time-invariant | - | 53.5 | 1.1 (1.0 - 1.2) | 1.1 (1.0 - 1.2) | 1.0 (1.0 - 1.2) | 1.1 (1.0 - 1.2) |
|  | aPTT (sec) | Time-invariant | - | 56.6 | 33.7 (30.0 - 40.0) | 33.8 (30.0 - 40.1) | 33.4 (29.8 - 39.7) | 33.5 (29.9 - 39.8) |
|  | Blood urea nitrogen (mg/dL) | Time-invariant | - | 4.0 | 39 (21 - 62) | 38 (20 - 61) | 44 (22 - 66) | 41 (21 - 63) |
|  | Creatinine (mg/dL) | Time-invariant | - | 4.4 | 8.26 (5.69 - 10.54) | 8.46 (5.71 - 10.69) | 8.02 (5.76 - 10.19) | 7.7 (5.46 - 10.09) |
|  | Sodium (mmol/L) | Time-invariant | - | 4.5 | 137 (135 - 139) | 137 (135 - 139) | 137 (135 - 139) | 137 (135 - 139) |
|  | Potassium (mmol/L) | Time-invariant | - | 4.6 | 4.6 (4.1 - 5.2) | 4.6 (4.1 - 5.2) | 4.6 (4.1 - 5.1) | 4.6 (4.0 - 5.2) |
|  | Total CO2 (mmol/L) | Time-invariant | - | 8.6 | 24 (22 - 27) | 24 (22 - 27) | 24 (21 - 27) | 24 (22 - 27) |
|  | Chrloide (mEq/L) | Time-invariant | - | 4.6 | 97 (95 - 100) | 97 (95 - 100) | 98 (95 - 101) | 98 (95 - 100) |
|  | Phosphorus (mg/dL) | Time-invariant | - | 4.1 | 4.4 (3.5 - 5.4) | 4.4 (3.5 - 5.4) | 4.4 (3.5 - 5.3) | 4.4 (3.4 - 5.5) |
|  | Calcium (mg/dL) | Time-invariant | - | 4.0 | 8.9 (8.3 - 9.4) | 8.9 (8.4 - 9.4) | 8.8 (8.2 - 9.3) | 8.8 (8.3 - 9.4) |
|  | Uric acid (mg/dL) | Time-invariant | - | 4.9 | 6.3 (5.0 - 7.7) | 6.4 (5.0 - 7.7) | 6.3 (5.0 - 7.7) | 6.1 (4.8 - 7.5) |
|  | CRP (mg/dL) | Time-invariant | - | 11.0 | 0.4 (0.1 - 2.4) | 0.4 (0.1 - 2.3) | 0.5 (0.1 - 3.1) | 0.4 (0.1 - 2.6) |
|  | Cystatin C (mg/dL) | Time-invariant | - | 95.9 | 4.4 (2.3 - 6.2) | 4.4 (2.3 - 6.2) | 4.6 (2.7 - 6.1) | 4.2 (2.4 - 6.0) |
|  | Glucose (mg/dL) | Time-invariant | - | 6.9 | 117 (92 - 160) | 118 (92 - 162) | 116 (91 - 148) | 113 (89 - 155) |
|  | LDH (IU/L) | Time-invariant | - | 85.5 | 250 (194 - 352) | 251 (193 - 355) | 245 (196 - 343) | 252 (195 - 348) |
| Medication | Antihypertensive drugs (%) | Time-invariant | - | 0 | 72.2 | 70.9 | 71.6 | 77.5 |
|  | Diuretics (%) | Time-invariant | - | 0 | 36.7 | 37.7 | 40.0 | 31.1 |
|  | Statin (%) | Time-invariant | - | 0 | 35.5 | 36.9 | 27.6 | 34.4 |
|  | Antiplatelet agents (%) | Time-invariant | - | 0 | 50.6 | 54.6 | 45.6 | 37.8 |
|  | Anticoagulants (%) | Time-invariant | - | 0 | 8.4 | 9.2 | 6.2 | 6.2 |
|  | Oral hypoglycemic agents (%) | Time-invariant | - | 0 | 11.7 | 13.5 | 8.9 | 6.4 |
|  | Subcutaneous insulin (%) | Time-invariant | - | 0 | 25.6 | 27.1 | 24.2 | 20.9 |
|  | Erythropoiesis-stimulating agents (%) | Time-invariant | - | 0 | 76.1 | 75.8 | 76.0 | 77.3 |
|  | Uric acid lowering agents (%) | Time-invariant | - | 0 | 38.5 | 39.5 | 33.3 | 37.5 |
|  | Phosphate binders (%) | Time-invariant | - | 0 | 73.1 | 73.9 | 73.4 | 69.6 |
| Study outcomes | IDH-1 | Time_varying | - | 0 | 10.7 | 10.8 | 11.5 | 10.2 |
|  | IDH-2 | Time_varying | - | 0 | 51.9 | 51.5 | 50.5 | 53.8 |
|  | IDHTN | Time_varying | - | 0 | 40.5 | 40.5 | 42.3 | 39.4 |

*Expression format in statistics: Binary or categorical features, percentage; continuous features with normal distribution, mean ± standard deviation; continuous features without normal distribution: median (25%-75% interquartile range).

**Supplementary Table 2. Precision, recall, and F1 scores for predicting intradialytic hypotension and hypertension.**

| Outcomes | Models | Precision | Recall | F1 score |
| --- | --- | --- | --- | --- |
| IDH-1 | TFT-based | 0.562 | 0.718 | 0.630 |
|  | RNN | 0.363 | 0.731 | 0.485 |
|  | LightGBM | 0.374 | 0.745 | 0.498 |
|  | Random forest | 0.309 | 0.783 | 0.443 |
|  | Logistic regression | 0.335 | 0.717 | 0.457 |
| IDH-2 | TFT-based | 0.701 | 0.778 | 0.738 |
|  | RNN | 0.630 | 0.772 | 0.694 |
|  | LightGBM | 0.624 | 0.772 | 0.690 |
|  | Random forest | 0.407 | 0.845 | 0.550 |
|  | Logistic regression | 0.593 | 0.769 | 0.670 |
| IDHTN | TFT-based | 0.592 | 0.766 | 0.668 |
|  | RNN | 0.544 | 0.758 | 0.634 |
|  | LightGBM | 0.542 | 0.746 | 0.628 |
|  | Random forest | 0.281 | 0.843 | 0.421 |
|  | Logistic regression | 0.515 | 0.742 | 0.608 |

**Supplementary Table 3. Model performance for predicting intradialytic hypotension and extendedly defined intradialytic hypertension.**

| Outcomes | AUROC (95% CI) | AUPRC (95% CI) | Recall | Precision | F1 |
| --- | --- | --- | --- | --- | --- |
| IDH-1 | 0.952 (0.951–0.953) | 0.712 (0.710–0.713) | 0.479 | 0.765 | 0.589 |
| IDH-2 | 0.888 (0.887–0.889) | 0.833 (0.832–0.834) | 0.710 | 0.764 | 0.736 |
| IDHTN-2 | 0.917 (0.916–0.918) | 0.728 (0.727–0.730) | 0.558 | 0.722 | 0.629 |

**Supplementary Figure 1. Timestamp count histogram and the occurrence of IDH-1, IDH-2, and IDHTN according to dialysis time.**


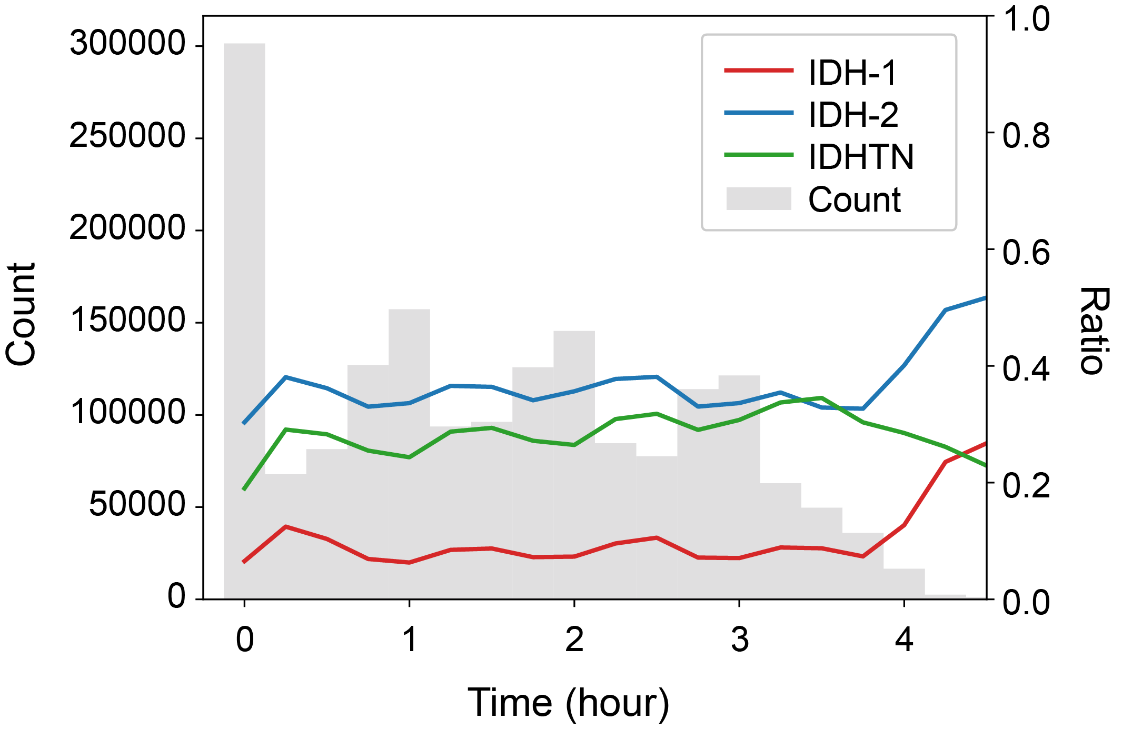


**Supplementary Figure 2. Calibration plot of the TFT-based model.**


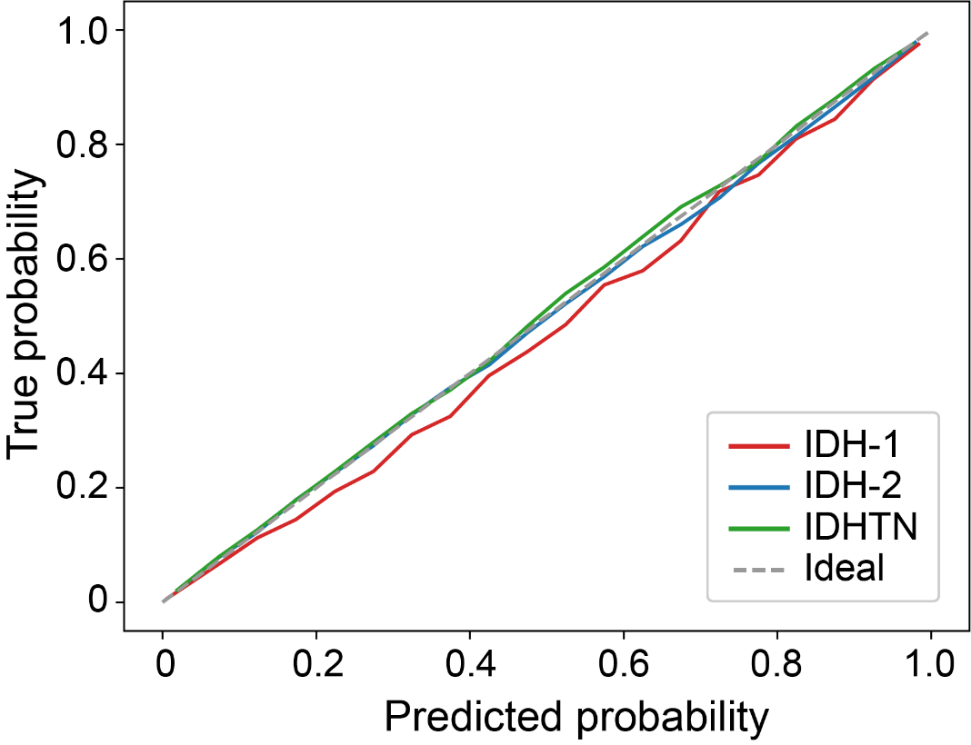

Supplement: Supplementary file 1 — Supplementary Information. [file 41598_2023_45282_MOESM1_ESM.docx]
